# Knowledge, attitude, and practice toward medical nutritional therapy among patients with chronic kidney disease

**DOI:** 10.3389/fnut.2026.1750998

**Published:** 2026-06-17

**Authors:** Dongxue Wang, Yali Liu, Shan Cong, Feng Xu

**Affiliations:** 1 Department of Pharmacy, The Second Hospital of Jilin University, Jilin, Changchun, China; 2 Department of Nephrology, The Second Hospital of Jilin University, Jilin, Changchun, China

**Keywords:** attitude, chronic kidney disease, knowledge, medical nutritional therapy, practice

## Abstract

**Introduction:**

Patients with chronic kidney disease (CKD) face challenges in managing their nutritional needs. Understanding the knowledge, attitude, and practice (KAP) regarding medical nutritional therapy among these patients is crucial for improving outcomes. This study aimed to investigate the KAP regarding medical nutritional therapy among patients with CKD.

**Methods:**

A cross-sectional study was conducted between September 1, 2022 and September 1, 2023 among patients with CKD. Patients’ KAP concerning medical nutritional therapy were assessed, and structural equation modeling (SEM) was utilized to analyze the interrelationships among these KAP dimensions.

**Results:**

A total of 481 valid questionnaires were analyzed, including 254 (52.81%) males, and 329 (68.40%) were in stage 5 CKD. The knowledge, attitude, and practice scores were 6.16 ± 2.85 (possible range: 0–12), 31.68 ± 3.96 (possible range: 8–40), and 35.6 ± 9.99 (possible range: 11–55), respectively. Spearman correlation analysis revealed significant positive correlations between knowledge and attitude (*r* = 0.426, *p* < 0.001), knowledge and practice (*r* = 0.422, *p* < 0.001), as well as attitude and practice (*r* = 0.327, *p* < 0.001). SEM showed that knowledge was positively associated with attitude (*β* = 0.907, *p* = 0.005) and practice (*β* = 1.482, *p* = 0.012).

**Conclusion:**

Patients with CKD exhibited inadequate knowledge, positive attitude, and passive practice. Targeted educational interventions are essential to enhance patients’ understanding of medical nutritional therapy for CKD.

## Introduction

Chronic Kidney Disease (CKD) has a high global prevalence, estimated at 11%–13%, with China experiencing notably elevated rates at 10.8% (1, 2). In recent years, there has been a concerning rise in CKD cases, resulting in 700 million CKD patients and 1.2 million CKD-related deaths in 2017. CKD now holds the top position in both disability and fatality rates among chronic diseases (3, 4). CKD patients not only contend with anorexia or insufficient nutrient intake due to uremic symptoms but also grapple with inflammatory conditions and oxidative stress, which significantly elevate the risk of malnutrition (5). In this context, dietary modifications and the maintenance of adequate nutrient homeostasis constitute a foundational strategy in the management of patients with CKD (6, 7). Addressing these nutritional complexities is of paramount importance in the holistic care and treatment of CKD patients.

Knowledge, Attitudes, and Practices (KAP) surveys are commonly used to assess a population’s understanding, beliefs, and behaviors regarding a specific health-related topic. This survey operates on the premise that knowledge plays a constructive role in shaping attitudes, which in turn mold behaviors (8–10). CKD not only affects renal function but also triggers various metabolic and physiological changes, leading to complications such as malnutrition, muscle wasting, and metabolic imbalances. Effective nutritional therapy plays a pivotal role in ameliorating these complications and promoting optimal patient outcomes. It encompasses dietary modifications, protein and energy intake adjustments, and management of specific nutrients to alleviate the burden of CKD-related symptoms and slow disease progression. Given the profound impact of CKD on patients’ quality of life and overall health, coupled with the frequently imbalanced nutritional status that tends to develop during the course of CKD, the significance of nutritional intervention in CKD patient management cannot be overstated (11, 12).

Understanding patients’ levels of KAP regarding medical nutritional therapy is crucial for improving their care and treatment. Although previous KAP studies have examined CKD-related knowledge and practices, limited evidence is available regarding patients’ KAP specifically related to medical nutritional therapy, including protein intake, protein quality, dietary assessment, and individualized dietary management (13, 14). Moreover, it remains unclear whether positive attitudes toward medical nutritional therapy are accompanied by corresponding dietary practices among patients with CKD. Therefore, this study aimed to investigate the KAP regarding medical nutritional therapy among patients with CKD and to explore the associations among knowledge, attitude, and practice in this specific nutritional management context.

## Materials and methods

### Study design and participants

This cross-sectional study was conducted between September 1, 2022 and September 1, 2023 among patients with CKD. Participants were recruited using consecutive sampling from the Nephrology Department of the Second Hospital of Jilin University. All eligible patients who visited the outpatient clinic or were admitted to the inpatient department during the study period were invited to participate until the target sample size was reached. The inclusion criteria were: 1) Age ≥ 18 years; 2) They must have a diagnosis of CKD stages 1–5, following the 2020 Kidney Disease: Improving Global Outcomes (KDIGO) guidelines, including those on renal replacement therapy or with a history of kidney transplantation (15); 3) Participants must be able to understand the study’s objectives, provide informed consent, and independently complete assessments, including questionnaires and interviews. The exclusion criteria were: 1) Those with severe, uncontrolled comorbidities that may significantly affect nutritional status or participation; 2) Individuals with severe cognitive impairments that hinder reliable information provision or informed consent; 3) Participants currently involved in other clinical studies related to nutritional interventions.

This study was approved by the Ethic Committee of the Second Hospital of Jilin University (2023–129), and all participants provided informed consent.

### Questionnaire and quality control

The questionnaire design drew inspiration from the KDOQI Clinical Practice Guideline for Nutrition in CKD: 2020 Update (16), Expert Consensus on Protein Nutrition Therapy for Chronic Kidney Disease, and the Clinical Practice Guidelines for Nutritional Therapy in chronic kidney disease in China (17). The questionnaire items were developed through a systematic process of adapting established guidelines to our specific research context. For the knowledge dimension, we extracted key nutritional recommendations from the KDOQI guidelines and Chinese Clinical Practice Guidelines, focusing on protein intake, energy requirements, and nutrient management specific to CKD. These technical recommendations were then transformed into clear, assessable knowledge statements appropriate for patients. The attitude dimension was designed to assess patients’ perspectives on these same nutritional principles, converting clinical recommendations into statements measuring agreement or acceptance. The practice dimension was developed by identifying observable behaviors that would indicate adherence to guideline recommendations, such as food selection habits, portion control practices, and engagement with nutritional services. This systematic adaptation process ensured that the questionnaire comprehensively covered the essential aspects of medical nutritional therapy while remaining accessible to patients with varying levels of health literacy. Following the initial design phase, a pilot test was conducted on a small sample (41 copies) to assess its reliability, yielding an acceptable Cronbach’s alpha of 0.828. The Kaiser-Meyer-Olkin (KMO) measure of sampling adequacy was 0.912, demonstrating good sample adequacy for factor analysis. The comparative and incremental fit indices were IFI = 0.863, TLI = 0.851, and CFI = 0.862. Although these values were slightly lower than the conventional threshold of 0.90 for indicating good model fit, previous studies have also regarded CFI and TLI values above 0.80 as acceptable in exploratory questionnaire validation or applied factor analysis contexts (18). The relatively modest fit indices in the present study may be partly related to the multidimensional nature of the clinical nutrition questionnaire and the heterogeneity of patients’ cognition regarding medical nutritional therapy. Considering the exploratory nature of this survey in a specific CKD population, together with the good KMO value and acceptable Cronbach’s alpha, the questionnaire was considered to have acceptable preliminary reliability and construct validity at this stage.

The final questionnaire, written in Chinese, consists of four distinct dimensions (Questionnaire). The knowledge dimension comprises 12 questions, scored with 1 point for correct answers and 0 points for incorrect or unclear responses, resulting in a score range of 0–12 points. The attitudes dimension consists of 8 questions, employing a five-point Likert scale, ranging from “Very Positive” (5 points) to “Very Negative” (1 point), yielding a score range of 8–40. The Practice Dimension comprises 8 questions and 11 items, also using a five-point Likert scale to assess the degree of positive behavior, ranging from “Always” (5 points) to “Never” (1 point). The score ranges from 11 to 55. Attaining scores above 70% of the maximum in each section indicates adequate knowledge, a positive attitude, and proactive practice. This 70% threshold was adopted based on previous similar KAP studies in healthcare settings (19, 20), as it provides a practical and commonly used criterion for distinguishing relatively sufficient from insufficient KAP levels in descriptive cross-sectional surveys. However, because this threshold has not been specifically validated among patients with CKD receiving medical nutritional therapy, it was used as a reference criterion rather than as a clinically validated diagnostic cutoff.

The survey was conducted using a combination of paper-based and electronic questionnaires. Paper-based questionnaires were distributed by members of the research team through the head nurse of the Nephrology Department, aiming to attract patients’ attention to the importance of the survey. The head nurse and research team members distributed and accompanied patients in person to complete the paper-based survey, and then collected the questionnaires face-to-face. Research team members simultaneously conducted quality control on the collected questionnaires, and only those meeting quality standards were accepted. Paper questionnaires were stored in locked cabinets, and electronic data were password-protected with access limited to the research team members. Personal identifiers were removed from the dataset prior to analysis, and results are reported only in aggregate form to protect participant privacy. The electronic version of the questionnaire was created using the Wenjuanxing (Questionnaire Star) app, and the head nurse of the Nephrology Department distributed it to the WeChat group of patients with chronic kidney disease. Research team members conducted quality control on the electronic questionnaires through the Wenjuanxing app. All participant data were anonymized during data collection and analysis to ensure confidentiality.

## Statistical analysis

Data analysis was conducted using SPSS 26.0 (IBM, Armonk, NY, United States), and structural equation modeling (SEM) was performed using SPSS AMOS version 26.0. Continuous data are presented as means and standard deviations (SD), while categorical data are expressed as n (%). The normality of continuous variables was assessed before group comparisons. For comparisons between two groups, the *t*-test was used for normally distributed data, and the Wilcoxon Mann–Whitney test was used for non-normally distributed data. For comparisons among three or more groups with normally distributed continuous variables and homogeneous variances, Analysis of Variance (ANOVA) was used. Homogeneity of variance was examined before ANOVA. Post-hoc tests with Bonferroni correction were conducted for multiple comparisons. For data not adhering to a normal distribution, the Kruskal-Wallis test was utilized. Spearman correlation analysis was employed to assess relationships among KAP. SEM was utilized to explore the relationships between knowledge (K), attitude (A), and practice (P), as SEM is commonly used in medical and health-related research to examine complex relationships among observed or latent variables. In the present study, knowledge, attitude, and practice were specified as observed variables represented by their total dimension scores, and no separate latent measurement model was constructed. Based on the theoretical framework of KAP and previous dietary KAP studies using SEM, the structural model specified paths from knowledge to attitude, from knowledge to practice, and from attitude to practice. The parameters were estimated using the maximum likelihood method. Model fit was evaluated using multiple commonly reported fit indices, including the chi-square/degrees of freedom ratio (*χ^2^*/df), root mean square error of approximation (RMSEA), goodness-of-fit index (GFI), incremental fit index (IFI), Tucker–Lewis index (TLI), and comparative fit index (CFI). In general, *χ*^2^/df < 3, RMSEA ≤ 0.08, and GFI, IFI, TLI, and CFI values close to or above 0.90 were considered indicative of acceptable model fit (21, 22). A two-sided *p*-value less than 0.05 was considered statistically significant.

## Results

### Demographic characteristics

**Figure 1:**
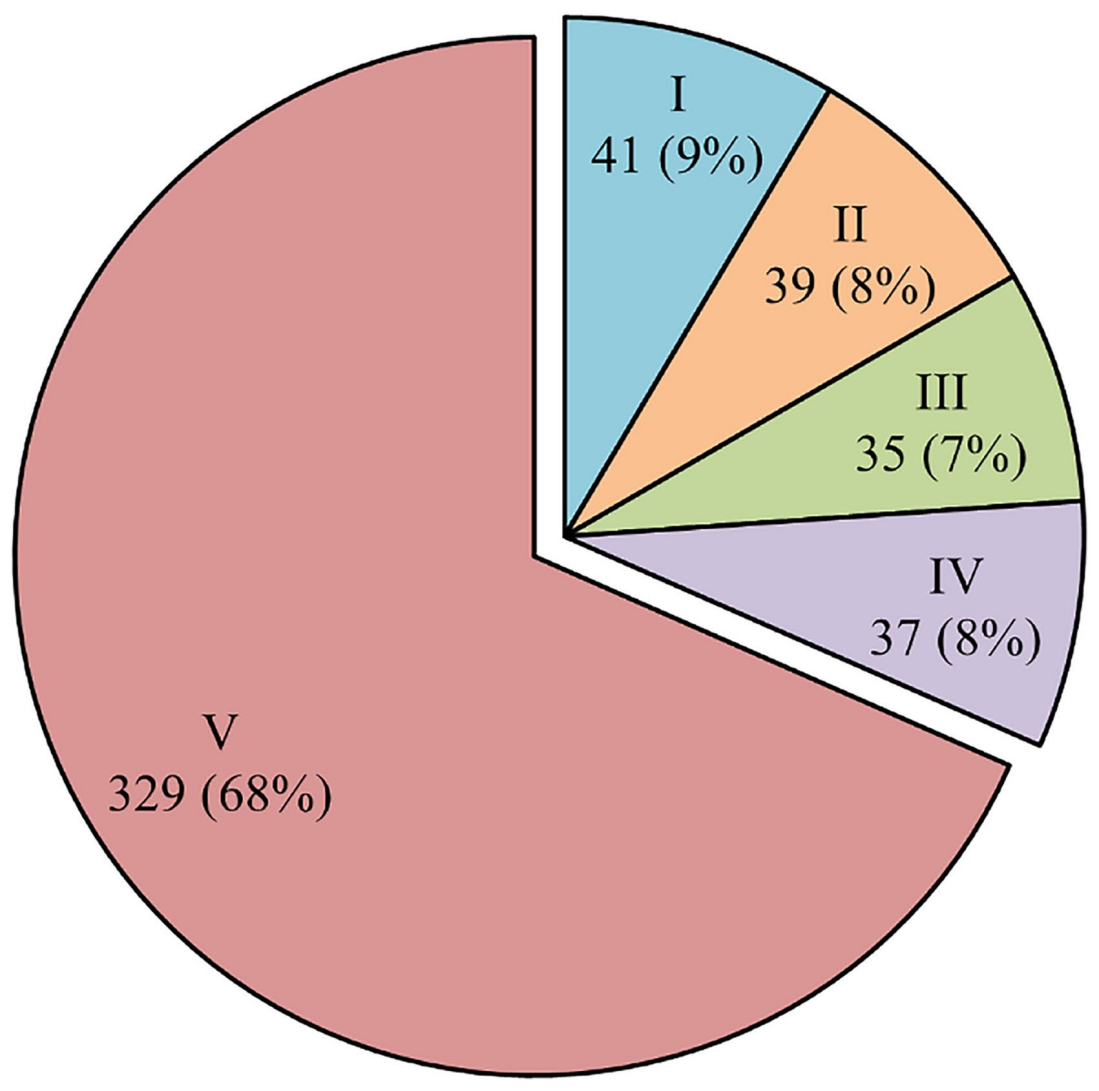
Staging of chronic kidney disease.

A total of 481 valid questionnaires were analyzed, yielding a validity rate of 96.98%. Among the respondents, 254 (52.81%) identified as male, while 329 (68.40%) were classified as being in stage 5 CKD (Figure 1). The predominant age group was 41–55 years, which constituted 31.60% of the participants. The duration of CKD among the participants was 81.80 ± 80.68 days. The knowledge, attitude, and practice scores were 6.16 ± 2.85 (possible range: 0–12), 31.68 ± 3.96 (possible range: 8–40), and 35.67 ± 9.99 (possible range: 11–55), respectively. Significant variations were identified in the examination of the influence of demographic and clinical characteristics on the scores. Knowledge scores exhibited notable differences based on monthly per capita income (*p* = 0.011), with patients in higher income brackets displaying a greater understanding of medical nutritional therapy. Attitude scores were significantly correlated with several factors, including gender (*p* = 0.028), education level (*p* = 0.010), employment status (*p* = 0.030), monthly per capita income (*p* = 0.003), and current treatment status (*p* = 0.004). Furthermore, practice scores were significantly correlated with current treatment status (*p* = 0.002), with patients undergoing renal replacement therapy achieving higher mean practice scores (Table 1).

**Table 1:** Baseline characteristics and KAP Scores.

Variables	*N* (%)	Knowledge		Attitude		Practice	
		Mean ± SD	*p*	Mean ± SD	*p*	Mean ± SD	*p*
Total	481	6.16 ± 2.85		31.68 ± 3.96		35.67 ± 9.99	
Gender			0.156		0.028		0.147
Male	254 (52.81)	6.34 ± 2.89		32.05 ± 4.06		36.30 ± 10.26	
Female	227 (47.19)	5.97 ± 2.79		31.26 ± 3.80		34.97 ± 9.66	
Age (years)			0.352		0.854		0.225
21–40	104 (21.62)	5.81 ± 2.94		31.85 ± 3.72		34.50 ± 10.05	
41–55	152 (31.60)	6.20 ± 3.04		31.80 ± 3.79		35.00 ± 9.80	
56–65	114 (23.70)	6.11 ± 2.70		31.46 ± 4.36		36.64 ± 10.76	
65 and above	111 (23.08)	6.50 ± 2.62		31.57 ± 4.00		36.70 ± 9.30	
Education			0.091		0.010		0.603
Primary school or below	73 (15.18)	5.64 ± 2.94		30.45 ± 4.36		36.25 ± 10.64	
High school/vocational	125 (25.99)	5.89 ± 2.95		31.53 ± 4.20		35.50 ± 9.54	
College	128 (26.61)	6.28 ± 2.70		31.73 ± 3.83		34.78 ± 9.72	
Bachelor’s degree or higher	155 (32.22)	6.54 ± 2.80		32.32 ± 3.54		36.28 ± 10.30	
Employment status			0.154		0.030		0.553
Employed	101 (21.00)	6.51 ± 2.74		32.44 ± 3.85		35.15 ± 10.22	
Unemployed	380 (79.00)	6.07 ± 2.87		31.47 ± 3.97		35.81 ± 9.94	
Monthly per capita income, RMB			0.011		0.003		0.122
<2,000	177 (36.80)	5.69 ± 2.96		31.15 ± 4.26		34.75 ± 10.22	
2,000–5,000	201 (41.79)	6.30 ± 2.88		31.57 ± 3.74		35.66 ± 9.98	
>5,000	103 (21.41)	6.71 ± 2.46		32.79 ± 3.65		37.29 ± 9.50	
Marital status			0.273		0.878		0.054
Unmarried	50 (10.40)	5.90 ± 2.92		31.90 ± 3.67		34.18 ± 10.66	
Married	357 (74.22)	6.29 ± 2.76		31.62 ± 3.92		36.32 ± 9.78	
Other	74 (15.38)	5.76 ± 3.20		31.77 ± 4.37		33.58 ± 10.33	
Duration of CKD	81.80 ± 80.68		0.214		0.434		0.545
Current status			0.393		0.004		0.002
Not on Dialysis	69 (14.35)	5.75 ± 2.83		31.23 ± 3.73		35.10 ± 9.26	
Undertaking renal replacement therapy	412 (85.65)	6.23 ± 2.85		31.75 ± 3.99		35.77 ± 10.12	
CKD stage							
1	41 (8.52)						
2	39 (8.11)						
3	35 (7.28)						
4	37 (7.69)						
5	329 (68.40)						

Multiple comparisons	Knowledge		Attitude	
	*p*	Bonferroni *p*	*p*	Bonferroni *p*
Education				
Primary school or below VS High school/vocational	0.088	0.528		
Primary school or below VS College	0.046	0.274		
Primary school or below VS Bachelor’s degree or higher	0.003	0.017		
High school/vocational VS College	0.739	0.999		
High school/vocational VS Bachelor’s degree or higher	0.150	0.899		
College VS Bachelor’s degree or higher	0.272	0.999		
Monthly per capita income, RMB				
<2,000 VS 2000–5,000	0.028	0.083	0.366	0.999
<2,000 VS >5,000	0.008	0.024	0.001	0.004
2000–5,000 VS >5,000	0.397	0.999	0.011	0.032

#### Distribution of responses to knowledge, attitudes, and practice dimensions

Patient knowledge of medical nutritional therapy was mixed. While over two-thirds correctly identified high-quality protein sources (K5, 71.93%) and the need for vitamin/iron supplementation in very low-protein diets (K12, 69.44%), significant gaps existed. Fewer than 20% understood recommendations for moderate red meat consumption (K8, 18.71%) or the non-detrimental effect of soy on kidneys (K9, 12.89%). Critically, only 6.86% knew that ≥75% of protein intake should come from high-quality sources (K6) (Table 2).

**Table 2:** Responses of knowledge dimension.

Item	Correct rate *n* (%)
1. Individuals with stage 3–5 CKD or after Kidney transplants performedation should undergo routine nutritional screening at least every six months to identify the risk of protein-energy consumption.	317 (65.90)
2. Nutritional assessment should include but is not limited to appetite, dietary intake, body weight and Body Mass Index (BMI), biochemical data, anthropometric measurements, and nutrition-related medical examination results.	277 (57.59)
3. Protein intake is crucial for maintaining muscle in adults, but protein breakdown produces degradation products that require renal clearance.	333 (69.23)
4. A high-protein diet can increase the glomerular filtration load on the kidneys, potentially leading to glomerular sclerosis and hindering the excretion of metabolic waste products.	312 (64.86)
5. Meats, eggs, dairy, and soy-based foods contain essential amino acids necessary for the body, referred to as high-quality or complete proteins in the medical context.	346 (71.93)
6. At least 75% of protein intake should come from high-quality protein sources.	33 (6.86)
7. To avoid excessive protein intake or insufficient intake of high-quality proteins, individuals can consider opting for low-protein staple foods as alternatives to traditional staples.	276 (57.38)
8. Red meat is an excellent source of essential amino acids and important for zinc, iron, and vitamin intake; therefore, it should be consumed in moderation.	90 (18.71)
9. Soy and soy-based products contain a high amount of non-essential amino acids, which can exacerbate kidney damage and are not recommended for patients.	62 (12.89)
10. With sufficient protein intake, plant-based and animal-based diets have similar effects on nutritional status and do not lead to malnutrition.	275 (57.17)
11. For those primarily consuming plant-based protein, it’s essential to be vigilant about the risk of deficiencies in essential amino acids, vitamin B12, n-3 polyunsaturated fatty acids, iron, zinc, and regularly monitor electrolyte changes.	310 (64.45)
12. Patients on a very low-protein diet should consider additional supplementation of vitamins and iron supplements.	334 (69.44)

Attitudes towards medical nutritional therapy were generally positive. A majority recognized the importance of adequate energy intake (A2, 85.24%) and believed protein intake should be clinician-determined rather than self-restricted (A4, 86.9%). However, 65.69% still supported universal protein restriction for all CKD patients (A3), suggesting an oversimplified view. A strong desire for professional support was evident, with 84.2% finding protein calculation challenging and wishing for dietitian-provided recipes (A6), and 87.11% seeking more educational information from healthcare providers (A8) (Table 3).

**Table 3:** Responses of attitude dimension.

Item	Strongly agree	Agree	Neutral	Disagree	Strongly disagree
1. I am well-informed about the principles of eating.	168 (34.93)	192 (39.92)	112 (23.28)	6 (1.25)	3 (0.62)
2. Ensuring adequate energy intake is equally important as a low-protein, high-quality protein diet.	194 (40.33)	216 (44.91)	58 (12.06)	11 (2.29)	2 (0.42)
3. All CKD patients should restrict their protein intake.	141 (29.31)	175 (36.38)	93 (19.33)	70 (14.55)	2 (0.42)
4. It’s important to calculate the daily protein intake under the guidance of a doctor, rather than controlling it blindly.	180 (37.42)	238 (49.48)	55 (11.43)	7 (1.46)	1 (0.21)
5. In addition to dietary control, it’s also important to ensure sufficient energy intake to avoid malnutrition or physical weakness.	194 (40.33)	228 (47.40)	48 (9.98)	10 (2.08)	1 (0.21)
6. The method for calculating protein intake is too complex. I wish I could receive professional recipes regularly developed by dietitians.	185 (38.46)	220 (45.74)	66 (13.72)	8 (1.66)	2 (0.42)
7. By adhering to a scientifically designed diet, I believe we can slow down the progression of kidney disease and coexist harmoniously with kidney disease.	191 (39.71)	230 (47.82)	44 (9.15)	14 (2.91)	2 (0.42)
8. I wish to receive educational information from doctors and gain more knowledge about medical nutritional therapy.	203 (42.20)	216 (44.91)	47 (9.77)	13 (2.70)	2 (0.42)

Patient medical nutritional therapy practices indicated a passive approach with significant room for improvement. High-quality proteins were incorporated with varying frequency, though soy product use was less common (P1). Adherence to specific dietary management tools was low, 36.59% never used a food scale (P2), 24.12% never reduced staple food intake (P3), and 30.56% never counted calories or protein per meal (P5). Conversely, most patients positively engaged in nutritional assessments (P6, 82.21% always/sometimes), sought personalized dietary counseling (P7, 76.93% always/sometimes), and utilized nutritional supplements (P8, 74.85% always/sometimes) (Table 4). Overall, the response patterns shown in Tables 2–4 indicate that patients generally had positive attitudes toward medical nutritional therapy, but their knowledge of protein quality and their use of practical dietary management tools remained limited.

**Table 4:** Responses of practice dimension.

Item	Always	Often	Sometimes	Rarely	Never
1. Incorporate high-quality proteins					
1.1 Lean meat (skinless)	142 (29.52)	158 (32.85)	133 (27.65)	34 (7.07)	14 (2.91)
1.2 Eggs	148 (30.77)	191 (39.71)	90 (18.71)	43 (8.94)	9 (1.87)
1.3 Dairy	113 (23.49)	117 (24.32)	95 (19.75)	112 (23.28)	44 (9.15)
1.4 Soy and soy-based products	83 (17.26)	100 (20.79)	141 (29.31)	128 (26.61)	29 (6.03)
2. Have a small food scale on hand	101 (21.00)	70 (14.55)	78 (16.22)	56 (11.64)	176 (36.59)
3. Reduce the consumption of common staples like rice and flours. Replace a portion of these staples with low-protein, pure starch foods like lotus root powder, wheat starch, and rice noodles.	79 (16.42)	68 (14.14)	131 (27.23)	87 (18.09)	116 (24.12)
4. Plan Your Three Meals Thoughtfully, distributing a variety of food types evenly across them.	101 (21.00)	123 (25.57)	120 (24.95)	63 (13.10)	74 (15.38)
5. Calculate the calories and protein intake for each meal.	84 (17.46)	76 (15.80)	90 (18.71)	84 (17.46)	147 (30.56)
Regarding the following statements, please indicate your willingness to implement them:					
6. Undergo regular nutritional assessments.	124 (25.48)	112 (23.28)	159 (33.06)	37 (7.69)	49 (10.19)
7. Seek personalized dietary counseling from a dietitian.	119 (24.74)	101 (21.00)	150 (31.19)	51 (10.60)	60 (12.47)
8. Use nutritional supplements as needed.	96 (19.96)	103 (21.41)	161 (33.47)	60 (12.47)	61 (12.68)

#### Spearman correlation analysis

Spearman correlation analysis revealed significant positive correlations between knowledge and attitude (*r* = 0.426, *p* < 0.001), knowledge and practice (*r* = 0.422, *p* < 0.001), and attitude and practice (*r* = 0.327, *p* < 0.001) (Table 5).

**Table 5:** Spearman correlation analysis of knowledge, attitude, and practices.

Variable	Knowledge	Attitude	Practice
Knowledge	1		
Attitude	0.426 (*p* < 0.001)	1	
Practice	0.422 (*p* < 0.001)	0.327 (*p* < 0.001)	1

#### SEM and mediation effect

**Figure 2:**
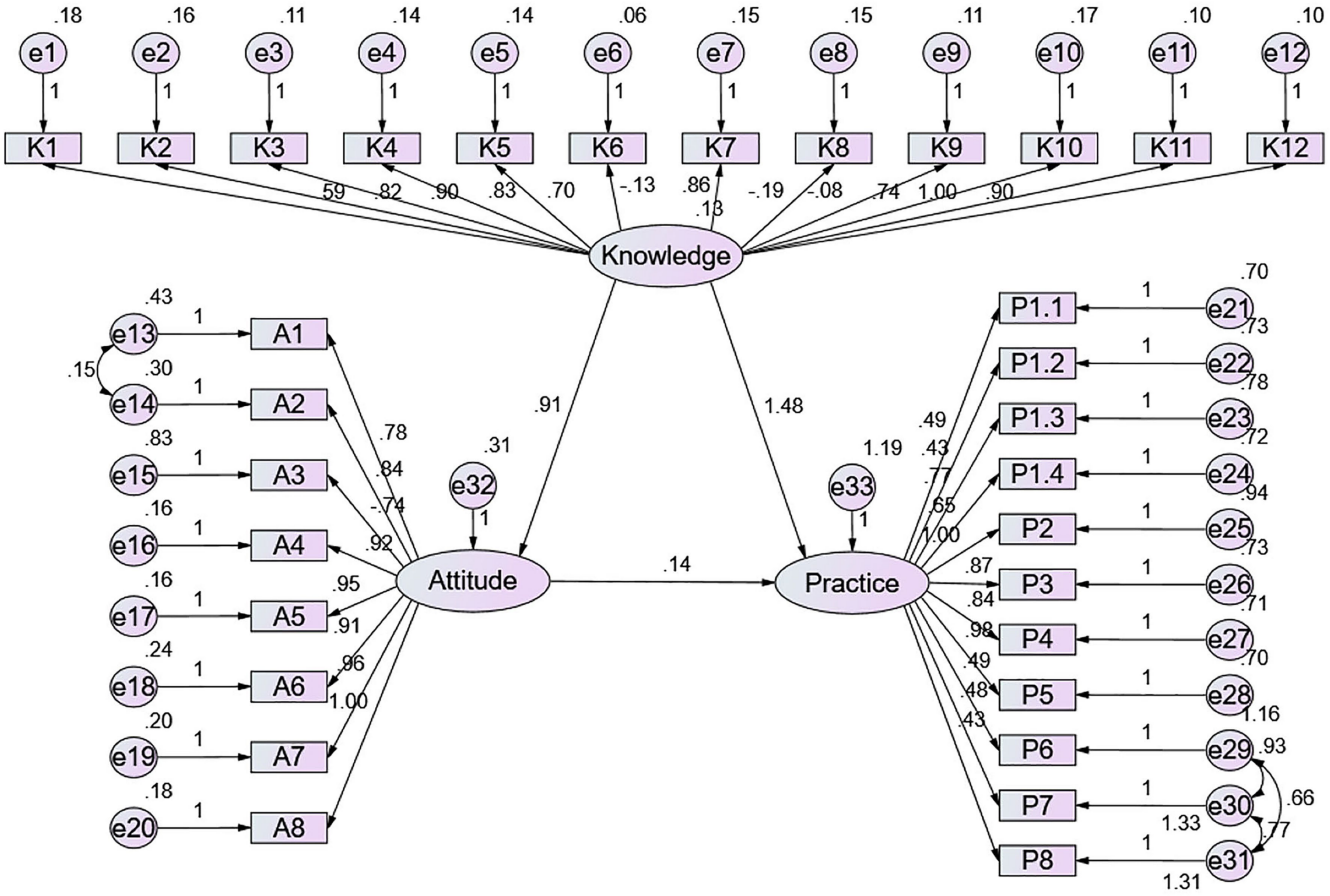
Model of structural equation modeling (SEM).

The SEM showed an acceptable model fit based on the fit indices reported in Supplementary Table S1. Knowledge was positively associated with attitude (*β* = 0.907, *p* = 0.005) and practice (*β* = 1.482, *p* = 0.012), whereas the association between attitude and practice was not statistically significant (*β* = 0.144, *p* = 0.242) (Figure 2, Table 6).

**Table 6:** Direct and indirect effect of structural equation modeling (SEM).

Model paths	Standardized total effects		Standardized direct effects		Standardized indirect effects	
	*β* (95% CI)	*p*	*β* (95% CI)	*p*	*β* (95% CI)	*p*
Knowledge → attitude	0.907 (0.700,1.960)	0.005	0.907 (0.700,1.960)	0.005		
Knowledge → practice	1.612 (1.271,1.960)	0.009	1.482 (1.079,1.939)	0.012		
Attitude → practice	0.144 (−0.080,0.383)	0.242	0.144 (−0.080,0.383)	0.242		
Knowledge → practice					0.130 (−0.073,0.355)	0.222

## Discussion

Patients with CKD exhibited insufficient knowledge, positive attitude, and passive practice regarding medical nutritional therapy. These findings suggest that although patients generally recognized the importance of medical nutritional therapy, gaps remained in their understanding and implementation of specific dietary recommendations.

Patients with elevated monthly per capita incomes exhibited superior knowledge scores, suggesting that socioeconomic status may be associated with access to nutritional information, educational resources, and overall health literacy. These factors may, in turn, be related to patients’ comprehension of medical nutritional therapy (23, 24). Additionally, various factors, including gender, educational attainment, employment status, monthly income, and current treatment status, influenced patient attitudes toward medical nutritional therapy. For instance, males, individuals with higher education levels, employed patients, and those with greater incomes generally demonstrated more favorable attitudes. These disparities suggest that personal background, educational achievement, and economic stability may be related to patients’ receptiveness to medical nutritional therapy, potentially through health literacy, exposure to diverse health information, or perceived ability to adhere to dietary recommendations (25, 26). In terms of reported dietary practice, only current treatment status was significantly associated with adherence, with patients undergoing renal replacement therapy displaying higher compliance with medical nutritional therapy guidelines. This may be attributable to their more frequent and intensive interactions with healthcare professionals, including dietitians, as part of their regular dialysis care. Such consistent clinical engagement likely reinforces adherence to medical nutritional therapy recommendations and provides ongoing support (27, 28). The lack of significant variation in practice scores across other demographic factors, particularly the difficulty in reliably assessing differences across CKD stages due to sample distribution, suggests that translating positive attitudes into consistent dietary practices may be complex. Possible barriers, such as financial constraints, cultural dietary habits, or limited access to specific foods, may contribute to this attitude-practice gap; however, these factors were not directly measured in the present study and should therefore be interpreted as possible explanations rather than confirmed mechanisms (29)These findings suggest that medical nutritional therapy education may need to be individualized and tailored to patients’ socioeconomic, educational, and treatment-related characteristics (30, 31). For instance, the integration of community-based nutrition programs and the provision of subsidized access to dietitians and low-protein food options could effectively mitigate the barriers encountered by low-income patients.

Notably, the research identified significant gaps in patients’ understanding of essential aspects of medical nutrition therapy. For instance, many participants were unaware of the importance of routine nutritional screening, which is critical for assessing the risk of protein-energy malnutrition. This lack of awareness regarding the significance of nutritional assessment components (such as appetite, dietary intake, and biochemical data) contradicts the comprehensive approach recommended in medical practice (16). Furthermore, misconceptions regarding protein intake, its sources, and the potential effects of a high-protein diet on kidney function underscore the necessity for targeted educational interventions. Additionally, misunderstandings about the dietary sources of essential and non-essential amino acids and their impact on kidney health indicate a pressing need for focused educational initiatives. Recent evidence also highlights the potential role of plant-based low-protein diets in improving nutritional and inflammatory status in patients with CKD, suggesting that dietary education should also address protein quality and protein source (32). These knowledge gaps underscore the need for targeted educational initiatives, particularly in the areas of routine nutritional screening, dietary assessment, and protein intake from both animal and plant sources. Such efforts will empower patients to make informed dietary choices and improve their self-management of CKD, aligning with previous research that emphasizes the critical role of patient education in managing CKD and promoting optimal nutritional practices (26, 33).

While many participants expressed a positive inclination to seek guidance from healthcare professionals and desired educational information from physicians, persistent misconceptions, such as the belief in a universal restriction on protein intake, were evident. Patients found the method for calculating protein intake to be complex and expressed a desire for professionally developed recipes. To enhance clinical practice, it is essential to address these deficiencies by offering clear and simplified dietary guidance, regularly providing professional recipes, and emphasizing the individualized nature of dietary management. These recommendations are consistent with prior findings that highlight the importance of tailored dietary interventions in improving patient outcomes (27, 28, 34). Additionally, healthcare providers should actively engage patients in discussions about dietary principles, the significance of balanced energy and protein intake, and the role of scientifically designed diets in managing kidney disease (29, 30).

The relationships among KAP in patients with CKD concerning medical nutritional therapy provide useful insights. Spearman correlation analysis and SEM showed positive associations between knowledge and attitude, knowledge and practice, and attitude and practice. In the SEM analysis, higher knowledge scores were associated with more positive attitudes and better reported practices, whereas the association between attitude and practice was not statistically significant. This finding suggests that additional factors may influence whether positive attitudes are translated into dietary practices. However, because barriers to dietary implementation were not directly measured, the present study cannot determine which specific factors explain this attitude-practice gap. Because this study was cross-sectional, these SEM findings should be interpreted as associations rather than evidence of causal relationships. Patients undergoing renal replacement therapy exhibited higher practice scores compared with those not yet on dialysis, possibly because they had more frequent healthcare interactions, including consultations with dietitians, as part of routine care. Future studies should explore how interactions during different treatment stages influence long-term KAP dynamics and help tailor interventions for pre-dialysis patients.

The study further reveals the practices of CKD patients regarding medical nutritional therapy, demonstrating a range of behaviors that indicate opportunities for improvement in clinical practice. Variability in the incorporation of high-quality proteins, limited use of small food scales, and challenges in reducing staple food consumption underscore the need for more consistent dietary management guidance. While some patients exhibit a willingness to undergo regular nutritional assessments, seek personalized dietary counseling, and utilize nutritional supplements, others may require additional support and motivation for consistent engagement in these practices. Initiatives aimed at enhancing clinical practice should prioritize patient education and support, offering clear guidance on protein intake, food scale usage, and structured meal planning, while actively involving patients in personalized dietary counseling and regular nutritional assessments (31, 35).

This study presents several limitations, particularly its cross-sectional design, which captures KAP at a singular point in time and restricts the ability to establish definitive causality or assess the temporal influence of knowledge on attitudes and practices. Although our SEM analysis indicates relationships among these variables, future longitudinal studies would be advantageous to confirm these pathways and elucidate how interventions might modify them. Additionally, this was a single-center study conducted in one hospital, which limits the external validity and generalizability of the findings to patients with CKD in other regions or healthcare settings. The sample was also heavily skewed toward patients with stage 5 CKD, who accounted for 68.40% of the participants. This sampling imbalance may have influenced the overall KAP results, as patients with advanced CKD or those receiving more frequent clinical care may have different levels of knowledge, attitudes, and practices compared with patients at earlier CKD stages. Moreover, the small number of patients in CKD stages 1–4 weakened the statistical power and reliability of subgroup comparisons across CKD stages. Therefore, the findings should be interpreted with caution and may not be fully generalizable to the broader CKD population across all disease stages. Self-administered questionnaires may introduce response bias, and reliance on self-reported practices may not accurately reflect patients’ actual dietary behaviors. In particular, patients may over report adherence to recommended dietary practices due to recall bias or social desirability bias. In addition, the 70% threshold used to categorize KAP levels was based on previous KAP studies and has not been specifically validated in patients with CKD receiving medical nutritional therapy; therefore, the categorized results should be interpreted as descriptive rather than diagnostic. Additionally, although the questionnaire showed an acceptable Cronbach’s alpha and a good KMO value, some model fit indices for construct validity were below the conventional threshold for good fit. Therefore, the questionnaire validity should be interpreted as preliminary, and further optimization and validation of the questionnaire in larger and more diverse CKD populations are needed. Furthermore, while we examined several socioeconomic factors, the study did not comprehensively analyze other potential cultural, psychological, behavioral, or environmental barriers that could influence the translation of positive attitudes into dietary practices. Future research should consider incorporating these variables and employing mixed-methods approaches for a more holistic understanding.

## Conclusion

In conclusion, patients with CKD demonstrated insufficient knowledge, positive attitudes, and passive practices. This study underscores the imperative to improve clinical approaches for CKD patients undergoing medical nutritional therapy. Despite positive attitudes, patients exhibited inadequate knowledge. These findings suggest the need for continued patient education, with attention to knowledge improvement and potential barriers between positive attitudes and dietary practices.

## Data Availability

The original contributions presented in the study are included in the article/Supplementary material, further inquiries can be directed to the corresponding author.
