# Supplementary material for: Knowledge, attitude, and practice toward medical nutritional therapy among patients with chronic kidney disease
Source: Front Nutr. 2026 Jun 17;13:1750998. doi: 10.3389/fnut.2026.1750998 (PMC13318730; doi:10.3389/fnut.2026.1750998)
Supplement: Supplementary file 1 [file Table_1.DOCX]

Table S1. Model Fit of KAP Model.

| **Indicator** | **Reference standard** | **Measured results** |
| --- | --- | --- |
| CMIN/DF | 1-3 excellent, 3-5 good | 2.660 |
| RMSEA | <0.08 good | 0.059 |
| IFI | >0.8 good | 0.902 |
| TLI | >0.8 good | 0.892 |
| CFI | >0.8 good | 0.901 |
