# Supplementary material for: Knowledge, attitude, and practice toward medical nutritional therapy among patients with chronic kidney disease
Source: Front Nutr. 2026 Jun 17;13:1750998. doi: 10.3389/fnut.2026.1750998 (PMC13318730; doi:10.3389/fnut.2026.1750998)
Supplement: Supplementary file 2 [file Table_2.DOCX]

**Knowledge, Attitude, and Practice toward Medical Nutritional Therapy among Patients with Chronic Kidney Disease**

| **Part I Basic Information** | | |
| --- | --- | --- |
| **1.Your age:** | years |  |
| **2.Your gender:** | a. Male  b. Female |  |
| **3.Your education:** | a. Primary school or below  b. High school/vocational  c. College  d. Bachelor's degree or higher | |
| **4.** **Employment Status** | a. Employed  b. Unemployed | |
| **5. Monthly per capita income (CNY)** | a. <2,000  b. 2,000-5,000  c. >5,000 | |
| **6.** **Marital Status** | a. Unmarried  b. Married  c. Other | |
| **7.** **Duration of CKD months** |  | |
| **8.** **Current Status** | a. Not on Dialysis  b. Peritoneal Dialysis  c. Hemodialysis  d. Kidney transplants performed | |
| **9.** **CKD Stage** | a. 1  b. 2  c. 3  d. 4  e. 5 | |

**Part Ⅱ Knowledge**

| 1. Individuals with stage 3-5 CKD or after Kidney transplants performedation should undergo routine nutritional screening at least every six months to identify the risk of protein-energy consumption. | a. Right | b. Wrong | c. Unclear |
| --- | --- | --- | --- |
| 2. Nutritional assessment should include but is not limited to appetite, dietary intake, body weight and Body Mass Index (BMI), biochemical data, anthropometric measurements, and nutrition-related medical examination results. | a. Right | b. Wrong | c. Unclear |
| 3. Protein intake is crucial for maintaining muscle in adults, but protein breakdown produces degradation products that require renal clearance. | a. Right | b. Wrong | c. Unclear |
| 4. A high-protein diet can increase the glomerular filtration load on the kidneys, potentially leading to glomerular sclerosis and hindering the excretion of metabolic waste products. | a. Right | b. Wrong | c. Unclear |
| 5. Meats, eggs, dairy, and soy-based foods contain essential amino acids necessary for the body, referred to as high-quality or complete proteins in the medical context. | a. Right | b. Wrong | c. Unclear |
| 6. At least 75% of protein intake should come from high-quality protein sources. | a. Right | b. Wrong | c. Unclear |
| 7. To avoid excessive protein intake or insufficient intake of high-quality proteins, individuals can consider opting for low-protein staple foods as alternatives to traditional staples. | a. Right | b. Wrong | c. Unclear |
| 8. Red meat is an excellent source of essential amino acids and important for zinc, iron, and vitamin intake; therefore, it should be consumed in moderation. | a. Right | b. Wrong | c. Unclear |
| 9. Soy and soy-based products contain a high amount of non-essential amino acids, which can exacerbate kidney damage and are not recommended for patients. | a. Right | b. Wrong | c. Unclear |
| 10. With sufficient protein intake, plant-based and animal-based diets have similar effects on nutritional status and do not lead to malnutrition. | a. Right | b. Wrong | c. Unclear |
| 11. For those primarily consuming plant-based protein, it's essential to be vigilant about the risk of deficiencies in essential amino acids, vitamin B12, n-3 polyunsaturated fatty acids, iron, zinc, and regularly monitor electrolyte changes. | a. Right | b. Wrong | c. Unclear |
| 12. Patients on a very low-protein diet should consider additional supplementation of vitamins and iron supplements. | a. Right | b. Wrong | c. Unclear |

| **Part III Attitude** | | | | | |
| --- | --- | --- | --- | --- | --- |
| 1. I am well-informed about the principles of eating. | a. Strongly agree | b. Agree | c. Neutral | d. Disagree | e. Strongly disagree |
| 2. Ensuring adequate energy intake is equally important as a low-protein, high-quality protein diet. | a. Strongly agree | b. Agree | c. Neutral | d. Disagree | e. Strongly disagree |
| 3. All CKD patients should restrict their protein intake. | a. Strongly agree | b. Agree | c. Neutral | d. Disagree | e. Strongly disagree |
| 4. It's important to calculate the daily protein intake under the guidance of a doctor, rather than controlling it blindly. | a. Strongly agree | b. Agree | c. Neutral | d. Disagree | e. Strongly disagree |
| 5. In addition to dietary control, it's also important to ensure sufficient energy intake to avoid malnutrition or physical weakness. | a. Strongly agree | b. Agree | c. Neutral | d. Disagree | e. Strongly disagree |
| 6. The method for calculating protein intake is too complex. I wish I could receive professional recipes regularly developed by dietitians. | a. Strongly agree | b. Agree | c. Neutral | d. Disagree | e. Strongly disagree |
| 7. By adhering to a scientifically designed diet, I believe we can slow down the progression of kidney disease and coexist harmoniously with kidney disease. | a. Strongly agree | b. Agree | c. Neutral | d. Disagree | e. Strongly disagree |
| 8. I wish to receive educational information from doctors and gain more knowledge about medical nutritional therapy. | a. Strongly agree | b. Agree | c. Neutral | d. Disagree | e. Strongly disagree |

| **Part IV Practice** | | | | | |  |
| --- | --- | --- | --- | --- | --- | --- |
| 1. Incorporate High-Quality Proteins |  |  |  |  |  | |
| 1.1 Lean meat (skinless) | a. Always | b. Often | c. Sometimes | d. Occasionally | e. Never | |
| 1.2 Eggs | a. Always | b. Often | c. Sometimes | d. Occasionally | e. Never | |
| 1.3 Dairy | a. Always | b. Often | c. Sometimes | d. Occasionally | e. Never | |
| 1.4 Soy and soy-based products | a. Always | b. Often | c. Sometimes | d. Occasionally | e. Never | |
| 2. Have a Small Food Scale on Hand. | a. Always | b. Often | c. Sometimes | d. Occasionally | e. Never | |
| 3. Reduce the consumption of common staples like rice and flours. Replace a portion of these staples with low-protein, pure starch foods like lotus root powder, wheat starch, and rice noodles. | a. Always | b. Often | c. Sometimes | d. Occasionally | e. Never | |
| 4. Plan Your Three Meals Thoughtfully, distributing a variety of food types evenly across them. | a. Always | b. Often | c. Sometimes | d. Occasionally | e. Never | |
| 5. Calculate the calories and protein intake for each meal. | a. Always | b. Often | c. Sometimes | d. Occasionally | e. Never | |
| Regarding the following statements, please indicate your willingness to implement them: |  |  |  |  |  | |
| 6. Undergo regular nutritional assessments. | a. Always | b. Often | c. Sometimes | d. Occasionally | e. Never | |
| 7. Seek personalized dietary counseling from a dietitian. | a. Always | b. Often | c. Sometimes | d. Occasionally | e. Never | |
| 8. Use nutritional supplements as needed. | a. Always | b. Often | c. Sometimes | d. Occasionally | e. Never | |
